# Overweight and obesity are associated with clustering of metabolic risk factors in early pregnancy and the risk of GDM

**DOI:** 10.1371/journal.pone.0225978

**Published:** 2019-12-03

**Authors:** I-Weng Yen, Chien-Nan Lee, Ming-Wei Lin, Kang-Chih Fan, Jung-Nan Wei, Kuan-Yu Chen, Szu-Chi Chen, Yi-Yun Tai, Chun-Heng Kuo, Chia-Hung Lin, Chih-Yao Hsu, Lee-Ming Chuang, Shin-Yu Lin, Hung-Yuan Li

**Affiliations:** 1 Department of Internal Medicine, National Taiwan University Hospital Hsin-Chu Branch, Hsin-Chu, Taiwan; 2 Department of Obstetrics and Gynecology, National Taiwan University Hospital, Taipei, Taiwan; 3 Chia Nan University of Pharmacy and Science, Tainan, Taiwan; 4 Ansn Clinic, Hsin-Chu, Taiwan; 5 Department of Internal Medicine, Cardinal Tien Hospital, New Taipei City, Taiwan; 6 Department of Internal Medicine, Fu Jen Catholic University Hospital, New Taipei City, Taiwan; 7 Department of Internal Medicine, National Taiwan University Hospital, Taipei, Taiwan; University of Mississippi Medical Center, UNITED STATES

## Abstract

**Aim:**

Overweight and obesity are important risk factors of gestational diabetes mellitus (GDM). Clustering of metabolic risk factors in early pregnancy may be a potential pathogenesis between the link of overweight/obesity and GDM. Since it remains unexplored, we investigated if overweight and obesity are associated with clustering of metabolic risk factors in early pregnancy and the risk of GDM in this cohort study.

**Methods:**

Total 527 women who visited National Taiwan University Hospital for prenatal care in between November 2013 to April 2018 were enrolled. Risk factors of GDM in the first prenatal visit (FPV) were recorded. Overweight/obesity was defined if body mass index ≥24 kg/m^2^. GDM was diagnosed from the result of a 75g oral glucose tolerance test in 24–28 gestational weeks.

**Results:**

Overweight/obesity was associated with clustering of metabolic risk factors of GDM, including high fasting plasma glucose, high HbA1c, insulin resistance, high plasma triglyceride and elevated blood pressure in FPV (*p*<0.05). There was a positive relationship between the number of metabolic risk factors and the incidence of GDM (*p* <0.05). The odds ratios of HbA1c and diastolic blood pressure were higher in overweight/obese women, compared with those in normal-weight women.

**Conclusions:**

Overweight/obesity is associated with clustering of metabolic risk factors in early pregnancy, which is correlated with higher risk of GDM. Our findings suggest that metabolic risk factors during early pregnancy should be evaluated in overweight/obese women.

## Introduction

Gestational diabetes mellitus (GDM) is defined when carbohydrate intolerance is developed or recognized during pregnancy for the first time [1]. GDM is prevalent among pregnant women. According to the report of International Diabetes Federation, the prevalence of GDM is about 14% worldwide in 2017 [2]. GDM results in increased risk of adverse pregnancy outcomes, including macrosomia, premature birth, hypoglycemia at birth, neonatal jaundice and congenital anomalies [3]. Furthermore, it is associated with a higher incidence of type 2 diabetes after delivery [4].

Obesity is an important risk factor of GDM [5]. Women with pre-pregnancy BMI over 30 have been shown to have a 3-fold increased risk of GDM, compared to women with normal weight before pregnancy [6]. Several mechanisms have been proposed, including elevated pro-inflammatory cytokines in maternal and fetal circulations and inflammation at placenta [7, 8]. In addition, clustering of metabolic abnormalities in obese women at early pregnancy may be another pathophysiology for the link between obesity and GDM. In non-pregnant status, metabolic abnormalities such as hypertension, central obesity, insulin resistance and atherogenic dyslipidemia tend to cluster. Clustering of these metabolic abnormalities is associated with the development of type 2 diabetes mellitus in the future [9]. During early pregnancy, clustering of these metabolic risk factors has been reported to correlate with increased risk of GDM [10]. Nonetheless, there is no report investigating the relationship among obesity, clustering of metabolic risk factors and GDM in early pregnancy.

In this cohort study, we enrolled 527 pregnant women and recorded their metabolic risk factors in early pregnancy. The relationship between overweight/obesity and clustering of metabolic risk factors were investigated. Moreover, the effect of clustering on the risk of GDM was explored.

## Materials and methods

### Subjects

We conducted a prospective cohort study, which recruited all the pregnant women having visited the obstetric clinic for prenatal care at National Taiwan University hospital obstetric clinic from November 2013 to April 2018. The pregnant women with overt diabetes, defined as diabetes diagnosed before pregnancy or at the first prenatal visit, and those with twin or multiple pregnancies, were excluded. The medical history, findings from physical examination, and results of laboratory tests of the participants were recorded at the first prenatal visit. All participants underwent a 75g oral glucose tolerance test (OGTT) at 24^th^–28^th^ gestational weeks to diagnose gestational diabetes. Body mass index (BMI) was calculated by body weight in kilograms divided by the square of body height in meters. Plasma glucose, hemoglobin A1c (HbA1c), total cholesterol (TC), high-density lipoprotein cholesterol (HDL-C), low-density lipoprotein cholesterol (LDL-C), plasma triglyceride (TG) and C-peptide were measured with an autonomic analyzer (Toshiba TBA 120 FR, Toshiba Medical Systems Co., Ltd., Tokyo, Japan). Written informed consent was obtained from each patient before enrollment in the cohort. This study was reviewed and approved by the Institutional Review Board of National Taiwan University Hospital.

### Definitions

Family history of diabetes was defined if one of the parents has diabetes. Overweight/obesity was defined as BMI ≥24 kg/m^2^. Updated computer models for homeostasis model assessment were used for calculation of HOMA2-IR. Insulin resistance was defined if HOMA2-IR of the subject was ≥ 25 percentile in this cohort. GDM was diagnosed according to the American diabetes association (ADA) criteria. Specifically, the diagnosis was made when any of the following criteria were met during an OGTT: 1). FPG ≥92 mg/dL (5.1 mmol/L); 2). 1-hour plasma glucose during OGTT (1hPG) ≥180 mg/dL (10.0 mmol/L); 3). 2-hour plasma glucose during OGTT (2hPG) ≥153 mg/dL (8.5 mmol/L).

### Statistical analysis

Data were presented as means and standard deviations for continuous variables, and as number and percentages for categorical variables. Student’s *t*-tests, Chi-squared tests and Fisher’s exact tests were used to identify the differences in clinical characteristics between the GDM and non-GDM groups. Linear regression were used to compare the difference in the clustering of risk factors between subjects with overweight/obesity and subjects with normal BMI. A logistic regression analysis was applied for the relationship between the number of risk factors and GDM, using GDM as the dependent variable. Logistic regression analyses were performed to identify important risk factors and to estimate their odds ratios of GDM. Interactions between risk factors and overweight/obesity on the risk of GDM were calculated. Statistical analyses were performed using Stata ⁄ SE 14.0 for Windows (StataCorp, College Station, TX, USA). The level of significance for all tests was p<0.05.

## Result

A total of 527 women were included in this study. The median gestational age of the first prenatal visit was 9.7 weeks (inter-quartile range 8.7–11 weeks). Their clinical characteristics in early pregnancy are shown in Table 1. The incidence rate of gestational diabetes was 12.6% in the normal weight group and 20.4% in the overweight/obesity group. Those developing gestational diabetes had older age, higher percentage with history of GDM and family history of diabetes, higher fasting plasma glucose, higher HbA1c, higher HOMA-IR (in the normal weight group), higher plasma triglyceride (in the normal weight group) and higher blood pressure (in the overweight/obesity group).

**Table 1:** Clinical characteristics of the study subjects in early pregnancy by gestational diabetes mellitus (GDM) and body mass index (BMI).

	BMI<24 kg/m2			BMI ≥24 kg/m2		
	Without GDM	With GDM	p	Without GDM	With GDM	p
N(%)	375 (87.4)	54 (12.6)		78 (79.6)	20 (20.4)	
Age (years)	33.5 ± 4.2	35.0 ± 4.1	0.038	34.2 ± 4.3	36.6 ± 3.3*	0.023
Gestational age at the first prenatal visit (week)	10.1 ± 1.9	10.1 ± 1.8	0.873	10.0 ± 1.8	10.0 ± 1.0	0.976
Parity (0/1/≥2, N, %)	235/118/21 (62.9/31.5/5.6)	23/21/5 (46.9/42.9/10.2)	0.074	33/35/10 (42.3/44.9/12.8)	8/10/2 (40/50/10)	0.940
Body weight before pregnancy (kg)	52.6 ± 5.5	53.0 ± 5.4	0.628	69.4 ± 8.4	72.6 ± 18.3	0.261
BMI before pregnancy (kg/m2)	20.3 ± 1.8	20.6 ± 1.7	0.333	27.4 ± 3.0	28.1 ± 6.7	0.489
History of GDM (N, %)	11 (2.9)	15 (27.8)	<0.001	3 (3.8)	5 (25.0)	0.008
History of preeclampsia (N, %)	5 (1.3)	1 (1.9)	0.556	1 (1.3)	2 (10.0)	0.105
History of PIH (N, %)	2 (0.5)	0 (0.0)	1.000	2 (2.6)	1 (5.0)	0.500
History of macrosomia (N, %)	2 (0.5)	1 (1.9)	0.333	1 (1.3)	0 (0)	1.000
Family history of DM (N, %)	84 (22.4)	20 (37.0)	0.019	16 (20.5)	10 (50.0)	0.008
At the first prenatal visit						
FPG (mg/dl)	81.4 ± 5.6	85.9 ± 7.5	<0.001	83.2 ± 5.7	88.0 ± 7.04	0.002
HbA1c (%)	5.3 ± 0.23	5.4 ± 0.37	0.002	5.3 ± 0.24	5.5 ± 0.27	<0.001
HOMA-IR†	0.64 ± 0.21	0.78 ± 0.27	0.038	1.06 ± 0.41	1.3 ± 0.42	0.083
Plasma total cholesterol (mg/dl)	175.3 ± 32.0	183.4 ± 42.0	0.106	180.5 ± 31.8	177.6 ± 36.7	0.737
Plasma triglyceride (mg/dl)†	100.5 ± 36.6	121.2 ± 48.9*	0.001	118.7 ± 47.2	126.0 ± 42.9	0.465
Plasma LDL-C (mg/dl)	91.5 ± 26.3	97.2 ± 32.5	0.160	100.7 ± 24.8	100.7 ± 25.1	0.996
Plasma HDL-C (mg/dl)	72.7 ± 14.6	70.1 ± 16.5	0.240	66.1 ± 14.0	59.9 ± 10.8	0.089
SBP (mmHg)	111 ± 11	112 ± 10	0.375	121 ± 13	130 ± 17	0.022
DBP (mmHg)	66 ± 9	66 ± 8	0.858	71 ± 9	78 ± 9	0.005

†statistical analyses were done after logarithmic transformation.

* p <0.05 vs. women without gestational diabetes in the same BMI subgroup

BMI, body mass index; GDM, gestational diabetes; PIH, pregnancy induced hypertension; DM, diabetes mellitus; FPG, fasting plasma glucose; LDL-C, low density lipoprotein cholesterol; HDL-C, high density lipoprotein cholesterol; SBP, systolic blood pressure; DBP, diastolic blood pressure.

As shown in Table 2, age, fasting plasma glucose, HbA1c, HOMA-IR, plasma triglyceride concentration and blood pressure at the first prenatal visit were significantly associated with the incidence of GDM (all p<0.05). In the overweight/obesity group, age, fasting plasma glucose, HbA1c, systolic blood pressure and diastolic blood pressure at the first prenatal visit were significant risk factors of GDM. Among all the risk factors, there were significant interactions for HbA1c, diastolic blood pressure, BMI group and GDM (both p<0.05). In other words, the odds ratios of GDM for HbA1c and diastolic blood pressure were significantly higher in overweight/obesity group than those in the normal weight group. Besides, plasma TG at FPV, but not other risk factors, was significantly associated with the gestational age at FPV (p<0.05). Therefore, we adjusted gestational age at FPV to explore the interaction between plasma TG at FPV, overweight/obesity and GDM. The result showed that the interaction was not statistically significant (p = 0.244). We performed linear regression for HbA1c and HOMA2-IR of these two groups, which showed: 1. Every 1% elevation of HbA1c increased HOMA2-IR 1.14 in normal weight group and 1.57 in overweight/obesity group. The p value of interaction between BMI and HbA1c to the influence of HOMA2-IR was 0.025, statistically significant. 2. Every 1 mmHg elevation of diastolic pressure increased HOMA2-IR 1.005 in normal weight group and 1.013 in overweight/obesity group. The p value of interaction between BMI and diastolic pressure to the influence of HOMA2-IR was 0.022, also statistically significant.

**Table 2:** Interaction of overweight and other factors at the first prenatal visit (FPV) on the risk of gestational diabetes (GDM). Odds ratios of every 1 standard deviation increase in risk factors for GDM were shown.

	BMI<24	BMI≥24	p value of the interaction
Age	1.36*	1.94*	0.289
FPG at FPV	2.02*	2.42*	0.720
HbA1c at FPV	1.60*	4.02*	0.025*
ln HOMA2-IR at FPV	1.78*	1.87	0.946
ln TG at FPV	1.64*	1.22	0.266
SBP at FPV	1.14	1.79*	0.213
DBP at FPV	1.03	2.26*	0.022*

FPG, fasting plasma glucose; HbA1c, hemoglobin A1c; ln, nature logarithm transformation; TG, plasma triglyceride; SBP, systolic blood pressure; DBP, diastolic blood pressure.

* p <0.05

**Fig 1:**
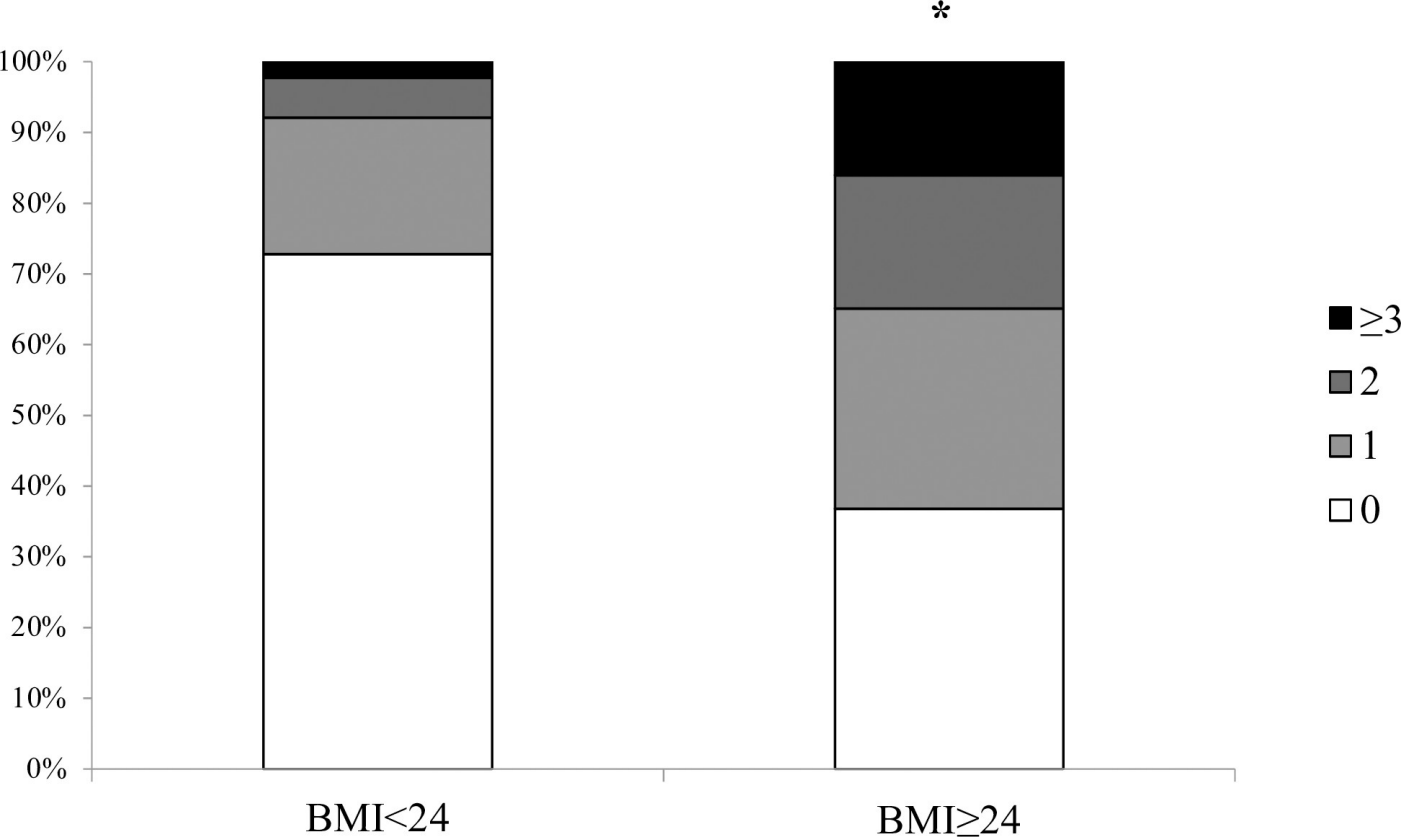
The proportion of different numbers of risk factors in early pregnancy in pregnant women with normal weight and overweight/obesity. Risk factors were measured in the first prenatal visit and included impaired fasting glucose (≥92mg/dl), HbA1c ≥5.7%, insulin resistance (HOMA2-IR ≥75 percentile), plasma triglyceride ≥150mg/dl and blood pressure ≥130/85mmHg. * p<0.05 vs. BMI<24.

Fig 1 shows the distribution of different numbers of risk factors in pregnant women with normal weight and overweight/obesity. Women in the overweight/obesity group tented to have more risk factors than those in the normal weight group (p<0.05), which means that risk factors are clustered in women with overweight/obesity. Further adjustment for gestational age resulted in similar finding (p<0.05)

**Fig 2:**
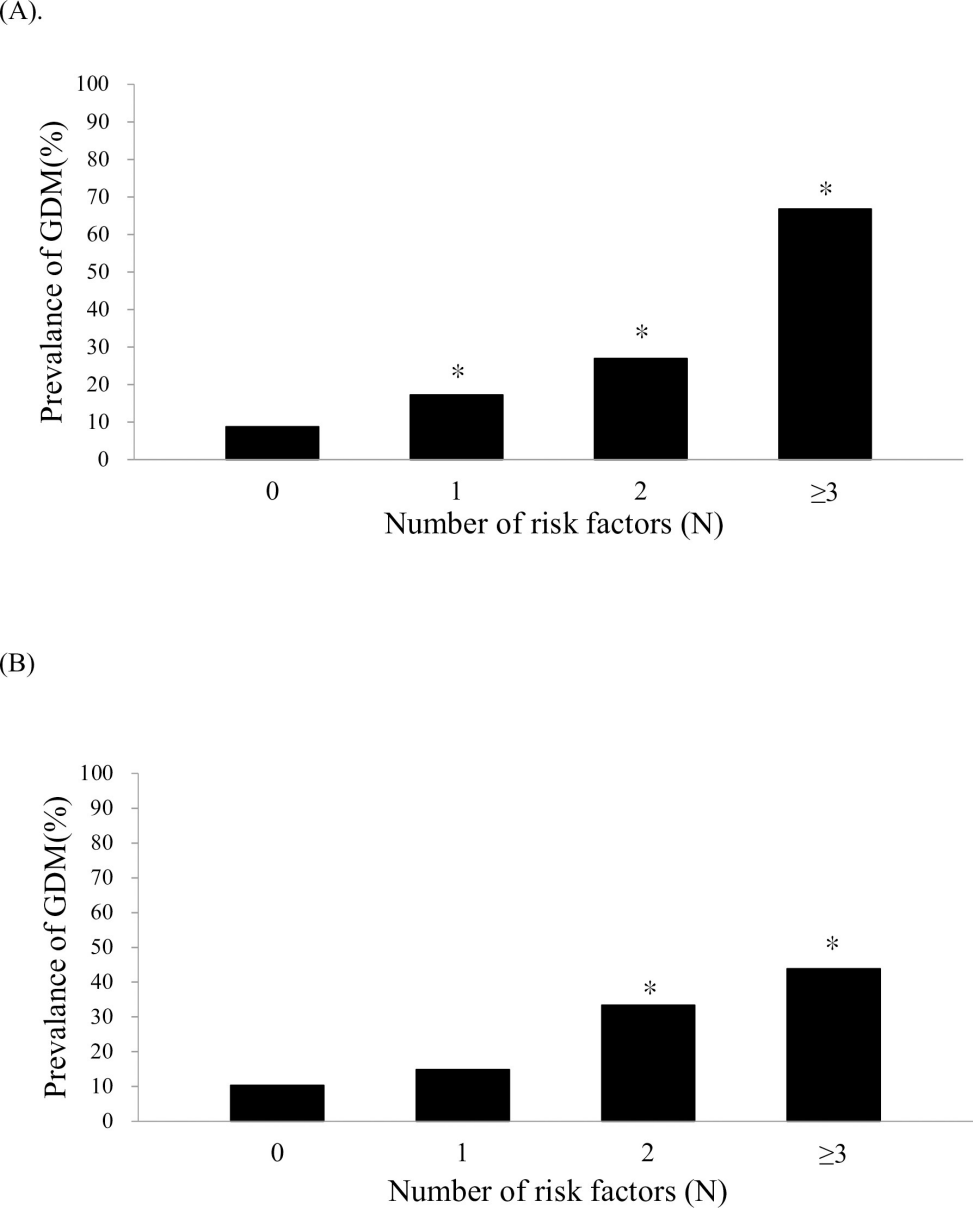
Number of risk factors in early pregnancy and the incidence of GDM. (A) normal weight group and (B) overweight/obesity group. * p<0.05 vs. 0 risk factor. P for trend <0.01.

Fig 2 shows the relationship between the numbers of risk factors in early pregnancy and the incidence of GDM in both normal weight and overweight/obesity group. In normal weight group, the incidence of GDM was 8.7% in women without any risk factor, 16.5% in women withone risk factor (p = 0.041 vs. women without risk factors), 28% in women with two risk factors (p<0.01 vs. women without risk factors) and 66.7% in women with more than three risk factors (p<0.01 vs. women without risk factors). In overweight/obesity group, the incidence of GDM was 10.5% in women without any risk factor, 11.5% in women who with risk factor (p = 0.899 vs. women without risk factors), 33.3% in women with two risk factors (p<0.05 vs. women without risk factors) and 43.8% in women with more than three risk factors (p<0.01 vs. women without risk factors). The incidence of GDM increased by the numbers of risk factors in the early pregnancy in both groups (normal weight group, p for trend<0.01, r^2^ = 0.9115; overweight/obesity group, p for trend<0.01, r^2^ = 0.8498) Further adjustment for gestational age showed similar results (p<0.01)

## Discussion

The major findings of this study are that metabolic risk factors in early pregnancy tend to cluster in pregnant woman with overweight/obesity, and clustering of these risk factors is associated with a higher risk of GDM. Our results suggest that clustering of metabolic abnormalities in overweight/obesity women in early pregnancy may be another pathophysiology for the link between overweight/obesity and GDM. The present study is the first report investigating the relationship among overweight/obesity, clustering of metabolic risk factors in early pregnancy and GDM. However, there are two articles including obesity as one of the metabolic risk factors and studying the relationship between clustering of these factors and GDM. In 2009, Chatzi et al. have described that metabolic syndrome can also be found during pregnancy [10]. They defined metabolic syndrome using the same criteria in non-pregnant people, except replacing waist circumference by BMI. Their findings supported the present study that obesity, higher blood pressure, fasting plasma glucose, HOMA2-IR and plasma triglyceride concentrations, and clustering of these factors in the first trimester were associated with higher risk of GDM. In another study, clustering of metabolic factors including obesity was associated with GDM as well as other adverse pregnancy outcomes [11].

In the present study, we found that obesity was associated with clustering of risk factors including insulin resistance, hypertriglyceridemia and elevated blood pressure during pregnancy. Obesity results in elevated plasma free fatty acid (FFA) levels, which leads to increased intracellular lipid accumulation in non-adipose cells, such as hepatocytes and skeletal muscle cells [12, 13]. Ectopic lipid accumulation in these cells can result in insulin resistance through the activation of protein kinase C and diacylglycerol pathways [14]. On the other hand, insulin can regulate plasma triglyceride concentrations by downregulation of microsomal triglyceride transfer protein (MTP) and activation of lipoprotein lipase (LDL) [15, 16]. In insulin resistant status, failure to inhibit MPL and activate LPL would lead to hypertriglyceridemia [17]. In addition, obesity can also activate renin-angiotensin-aldosterone system (RAAS), which is an important cause of hypertension[18]. Taken together, our findings are supported since obesity is a common cause of insulin resistance, hypertriglyceridemia and elevated blood pressure.

In this study, we found significant interactions between HbA1c, diastolic blood pressure and overweight/obesity on the risk of GDM. In other words, the odds ratio of HbA1c or diastolic blood pressure on GDM was significantly higher in pregnant women with overweight/obesity than those with normal weight. In the literature, HbA1c has been used to define high-risk group of GDM. One report in 2014 suggested the cutoff value to be set at 5.7%, and there was 30% of pregnant woman in their study group having BMI ≥30 kg/m^2^[19]. Our findings suggest that different cutoff values for HbA1c and blood pressure are needed to define high-risk group of GDM in women with overweight/obesity. However, the underlying pathophysiology for these interactions remains unclear. In our analyses, every unit increased in A1c and diastolic blood pressure was associated with a greater increase in HOMA2-IR in overweight/obese women, although the difference was borderline statistical significant due to the limited sample size. These findings suggest that with similar HbA1c or blood pressure, overweight/obese women were more insulin resistant than women with normal weight, which is a promising mechanism to be explored in the future.

This study is the first report which explored the relationship of overweight/obesity, clustering of metabolic risk factors and GDM with an adequate sample size. Our findings provide supporting evidence for clustering of metabolic risk factors as a pathogenic link between overweight/obesity and GDM. Besides, these metabolic risk factors were recorded at the first prenatal visit. Therefore, the temporal relationship between clustering of risk factors and GDM is clear. However, this study limited in the detail molecular mechanisms among overweight/obesity, clustering of metabolic risk factors and GDM were not explored, which should be studied in future studies.

In conclusion, the present study found that metabolic risk factors in early pregnancy tend to cluster in pregnant woman with overweight or obesity, and clustering of these risk factors is associated with a higher risk of GDM. Our findings suggest that clinicians should evaluate metabolic risk factors in early pregnancy, especially in obese or overweight pregnant women.
